# Biomechanical and clinical evaluation of 3D-printed integrated tibial prosthesis for reconstructing AORI type Ⅲ tibial plateau defects

**DOI:** 10.3389/fbioe.2025.1662741

**Published:** 2025-10-21

**Authors:** Yong Wang, Xiaoyu Zhou, Lin Guo

**Affiliations:** ^1^ Dalian Medical University, Dalian, China; ^2^ Department of Orthopedics, Affiliated Zhongshan Hospital of Dalian University, Dalian, China

**Keywords:** 3D-printed, integrated tibial prosthesis, finite element analysis (FEA), biomechanical evaluation, clinical efficacy, AORI classification

## Abstract

**Objective:**

To compare the biomechanical stability and clinical efficacy of 3D-printed integrated tibial prosthesis (ITP) and traditional modular augment prostheses (MAP) in reconstructing AORI Type Ⅲ tibial plateau defects, and to provide a reference for clinical decision-making.

**Methods:**

A finite element model of AORI Type Ⅲ tibial plateau defect (defect area >60%, depth >20 mm) was established using CT data of a healthy male subject. Four groups of models were constructed: Spacer, Cone, Sleeve, and ITP. Under different loads (700N, 1750N, 2100N, 2450N, simulating standing, knee flexion, stair climbing, and jogging), the contact stress at the prosthesis-bone interface, vertical displacement of the tibial plateau, and relative micromotion were analyzed. Additionally, a retrospective study was conducted on 6 patients with AORI Type Ⅲ defects who underwent TKA with ITP between January 2021 and January 2025, with clinical evaluation using KSS scores, X-ray imaging, and gait analysis.

**Results:**

Biomechanically, under all load conditions, ITP showed lower peak contact stress at the cortical bone, cancellous bone, and prosthesis interfaces (e.g., cortical bone stress at 2100N: 16.69 MPa for ITP vs 30.00 MPa for Spacer), smaller vertical displacement (245.6  μm at 2100N vs 385.2 μm for Spacer), and reduced relative micromotion (7.1  μm at 2100N vs 13.0 μm for Spacer). Clinically, the 24-month follow-up showed that the KSS score increased from 46.5 ± 4.8 preoperatively to 85.4 ± 5.5, with no loosening or osteolysis. Gait parameters (walking speed, step length, cadence) were significantly improved at 1 year postoperatively, and the affected side showed symmetry with the contralateral side. Due to the single-arm small sample size of the clinical cohort (n = 6) and the lack of a MAP control group, the clinical findings of this study are only preliminary observations.

## 1 Introduction

The knee joint, as a core structure for weight-bearing and movement in the human body, relies on the precise articulation between the tibial plateau and femoral condyles for stability. Pathological changes associated with tibial plateau defects often disrupt the anatomical alignment of the tibia and femur, leading to abnormal distribution of joint contact stress. This stress irregularity not only exacerbates mechanical instability of the knee but may also cause laxity in soft tissues such as cruciate and collateral ligaments, accelerating articular cartilage degeneration and ultimately progressing to end-stage osteoarthritis (Andriacchi et al., 2009). In the assessment of tibial plateau defects, the Anderson Orthopaedic Research Institute (AORI) classification system is widely used to guide surgical decision-making. Among its categories, AORI Type Ⅲ defects pose a clinical challenge due to significant metaphyseal bone loss and disruption of cortical bone integrity (Engh and Ammeen, 1998). Such defects not only make it difficult to reconstruct the joint line using conventional osteotomy techniques but also, due to the complexity of restoring lower limb alignment, are prone to complications such as malalignment and abnormal stress distribution (Clatworthy et al., 2001). Studies have shown that the postoperative prosthesis loosening rate in patients with AORI Type Ⅲ defects is as high as 18%, significantly higher than that in other types (Schroer et al., 2013). How to accurately restore the anatomical structure of the tibial plateau and reconstruct biomechanical stability has become a critical challenge in improving the efficacy of total knee arthroplasty (TKA) and revision total knee arthroplasty (rTKA).

Since the 1990s, customized tibial prosthesis components (such as wedges and cones) have been used in complex knee joint reconstruction, with their technical roots tracing back to the precision-machined customized prostheses of the 1950s (Jiao et al., 2023). Traditional techniques for reconstructing tibial plateau defects mainly include autogenous bone grafting, bone cement combined with screw reinforcement, metal augments, conical metal cones, and porous sleeves. Although these methods can fill bone defects and provide mechanical support in the short term, long-term follow-up data have revealed their limitations. For instance, autogenous bone grafting is associated with donor site complications and the risk of bone resorption, with a bone resorption rate as high as 40% at 5 years postoperatively (Engh and Ammeen, 2007). Although the bone cement-screw combination is easy to operate, the difference in elastic modulus between bone cement and host bone tends to cause stress shielding, leading to secondary fractures or prosthesis subsidence (Darwich et al., 2023). Although conical fillers, which have been widely used in recent years, can partially restore bone volume, their standardized design makes them difficult to adapt to complex bone defect morphologies. This often requires repeated adjustment of prosthesis position during surgery, increasing both operative time and infection risk (Denehy et al., 2019). Furthermore, the connection interfaces of modular components are prone to generating wear particles due to micromotion, which can induce aseptic loosening. A 10-year follow-up study on TKA revision surgery indicated that 23% of patients who received metal augments required secondary revision due to prosthesis loosening (Sharkey et al., 2014).

What is more noteworthy is that the core concept of traditional techniques relies on “defect filling” rather than “anatomical reconstruction,” which may result in imprecise restoration of the lower limb alignment. For example, excessive reliance on augments to elevate the joint line can alter the knee’s flexion-extension axis, exacerbating patellofemoral joint pressure (Figgie et al., 1989),Moreover, the rigid fixation of conical fillers may impede stress transmission to the host bone, accelerating micromotion at the prosthesis-bone interface (Small et al., 2022). The root cause of these issues lies in the inability of traditional methods to achieve individualized adaptation to defect morphology and dynamic balance of biomechanics.

With advancements in additive manufacturing technology, 3D printing has provided a new solution for the reconstruction of tibial plateau defects. Unlike traditional standardized prostheses—and by “traditional technology” we specifically refer to commercially available standardized Modular Augment Prostheses (MAP)—3D printing technology can accurately reconstruct a three-dimensional model of the patient’s bone defect using preoperative CT/MRI data (Guo et al., 2022), and design a matching integrated tibial prosthesis (Integrated Tibial Prosthesis, ITP). Compared with such MAP, the advantage of ITP lies in eliminating the connection interfaces between components, fundamentally avoiding the risks of micromotion, wear, and loosening (Mohan et al., 2013). Furthermore, the integrated design of the ITP simplifies surgical procedures and reduces intraoperative soft tissue dissection, making it particularly suitable for complex cases with severe osteoporosis or multiple revisions (Fram et al., 2020). However, systematic research on the biomechanical differences between ITP and MAP remains lacking, and experimental validation of their long-term reliability is urgently needed.

This study establishes an AORI Type Ⅲ tibial plateau defect model to simulate total knee arthroplasty (TKA), and comparatively analyzes the initial stability of tibial reconstruction using ITP and MAP under different loading conditions. It aims to provide a reference for clinically selecting appropriate tibial plateau defect reconstruction methods from a biomechanical perspective.

## 2 Materials and methods

### 2.1 Establishment of AORI type Ⅲ tibial plateau defect model

A 24-year-old healthy male (height 185 cm, weight 70 kg) was selected as the experimental subject. After he signed the informed consent form, a Siemens SOMATOM Definition AS scanner (128 Slice, 70 kV steps) was used to complete the knee joint scan. The DICOM-format tibial CT images were imported into Mimics 21.0. By setting appropriate grayscale values to distinguish the cortical bone and cancellous bone of the tibia, the three-dimensional model of the original knee joint was constructed. Subsequently, the model was imported into Geomagic Wrap 2021 for surface defect repair, smoothing, and precise optimization of surface functions.

### 2.2 Preoperative design and preparation of the prosthesis

After reconstructing the patient’s knee joint CT data on a computer and simulating the tibia to its true anatomical position, bone filler was placed in the tibial plateau defect to form an integrated structure matching autologous bone and the prosthetic tibia. Precise thickness, width, and height of the filler’s smooth curved transition were determined by biomechanical simulations, defect features, and residual bone mass.

To prevent stress shielding, the non-porous integrated tibial prosthesis (ITP) model was topologically optimized via Magics software (Materialise, Belgium) — adjusting internal mechanical paths and solid distribution to ensure structural stability and reduce stress concentration from redundant material.

Finally, the optimized ITP model was imported into an EOS M 290 metal 3D printer (EOS GmbH, Germany) for prosthesis fabrication using laser powder bed fusion. Equipped with a 400W fiber laser, the printer used a 30 μm layer thickness for precision. The material was ASTM F799-compliant cobalt-chromium-molybdenum alloy (UNS R31537: 63% Co, 28%–29% Cr, 6% Mo, plus trace Si, W), which offers excellent wear/corrosion resistance and mechanical strength, meeting long-term implantation needs.

### 2.3 Establishment and assembly of tibial bone defect model

**FIGURE 1:**
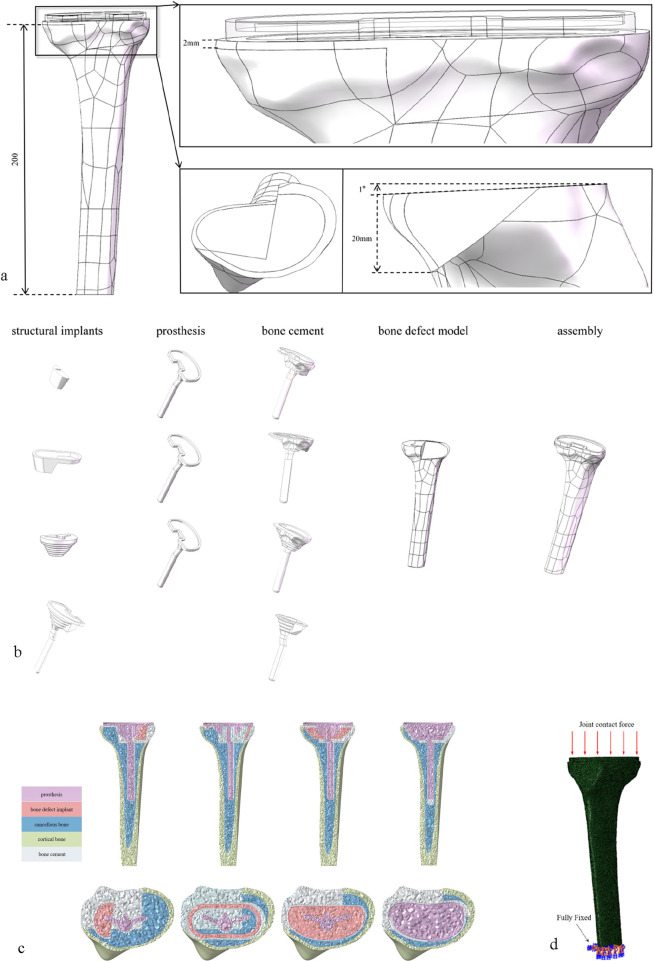
**(a)** A defect area of about 60%, a defect depth of about 20 mm and bone defect model with 1° caster angle. **(b)** The above-mentioned models refer to bone defect models repaired with block graft, cone graft, sleeve graft, and 3D-printed integrated prosthesis respectively. **(c)** All models were meshed with C3D4 tetrahedral elements. **(d)** Loading and boundary conditions of FE modeling.

Based on the anatomical parameters of the subject’s knee joint and with reference to a completed surgical case of tibial defect, a 200 mm tibial plateau defect model with a depth exceeding 20 mm and a defect area exceeding 60% was constructed in SOLIDWORKS (Figure 1a). In accordance with the principles of primary TKA (total knee arthroplasty), the tibial prosthesis implantation parameters were determined as follows: 0° varus (ensuring the proximal tibial resection is perpendicular to the tibial mechanical axis in the coronal plane), 1° posterior slope (the angle between the proximal tibial resection plane and the tibial anatomical axis in the sagittal plane), and 3° external rotation (the posterior edge line of the tibial tray rotates outward by 3° relative to the posterior edge line of the proximal tibial resection in the axial plane) (Dong et al., 2020). To simplify computational processes, modeling of the tibial liner, femoral component, and femoral stem was omitted; additionally, modeling of the ligaments and menisci was also excluded. Three-dimensional modeling and assembly of the knee prosthesis (including a 7 cm short stem), spacer, cone, sleeve, and 3D-printed integrated prosthesis were completed in SolidWorks (all prosthesis parameters were provided by JST Medical). A 2.0 mm-thick bone cement layer was used to fix the tibial tray and augments to the resected upper surface of the tibia, and the bone tissue area separated from the defect region was defined as the bone cement filling area (Figure 1b). The assembly was imported into Hypermesh, where the finite element model was meshed using C3D4 tetrahedral elements (Figure 1c). After exporting as an. hm file, it was imported into Abaqus for stress analysis. This finite element model was validated by comparison with *in vitro* mechanical test data from Zheng et al. (2020), with a contact stress error within 5%, ensuring its reliability (Zheng et al., 2020). Cortical bone and cancellous bone were fully bonded, and cancellous bone was fully bonded with the bone cement layer. The general contact algorithm was used to model the contact between bone and prosthesis, as well as between prosthesis and augments, with a constant friction coefficient of 0.3 applied to all models. In the finite element analysis, the femoral part of the prosthesis was simplified to a vertically downward load averaging on the plane of the tibial prosthesis. The tibia was truncated, and all degrees of freedom were fixed at a distance of 200 mm (Figure 1d).

### 2.4 Classification and material parameters of the finite element model

The finite element models were categorized into four groups based on component types: Group A (Spacer group), Group B (Cone group), Group C (Sleeve group), and Group D (Integrated prosthesis group). All models were meshed using C3D4 tetrahedral elements. To ensure geometric similarity and computational accuracy of the models, a global mesh size of 1 mm was set, and all materials were assumed to be continuous, isotropic, and homogeneous linear elastic bodies (Zheng et al., 2020). Table 1 details the mechanical parameters of each component in the model.

**TABLE 1:** Mechanical properties of different parts of the model.

Component	Material	Young’s modulus E (MPa)	Poisson’s ratio(v)	Number of element	Number of nodes
A	Cortical bone	17,000	0.3	396,968	86,728
B				398,687	87,083
C				397,873	86,869
D				396,299	86,615
A	Cancellous bone	700	0.3	263,829	56,317
B				268,903	57,734
C				225,984	49,602
D				247,106	52,885
Spacer	Cobalt-chrome alloy (tibial prothesis)	248,000	0.3	27,340	5,921
Cone				48,857	11,173
Sleeve				111,086	23,573
Prosthesis				118,601	26,515
Integrate prosthesis				153,992	33,018
A	PMMA (cement)	2,270	0.46	167,242	39,300
B				180,751	42,616
C				177,640	41,221
D				116,168	29,729

### 2.5 Setup of model parameters

The distal surface of the tibia was fully fixed in all directions. Based on the results of peak stress on the knee joint reported in relevant literature, the load when standing on both legs is 1 time the body weight (700N) (Northon et al., 2018),The load when the knee joint is flexed is 2.5 times the body weight (1750N) (Mesfar and Shirazi-Adl, 2005),The load when climbing stairs is 3 times the body weight (2100N) (Makani et al., 2022),The load during jogging is 3.5 times the body weight (2450N) (Miller et al., 2014). It is applied averagely on the tibial prosthesis platform.

### 2.6 Clinical application in repairing AORI type Ⅲ tibial plateau defects during knee arthroplasty

#### 2.6.1 Patient general information

**FIGURE 2:**
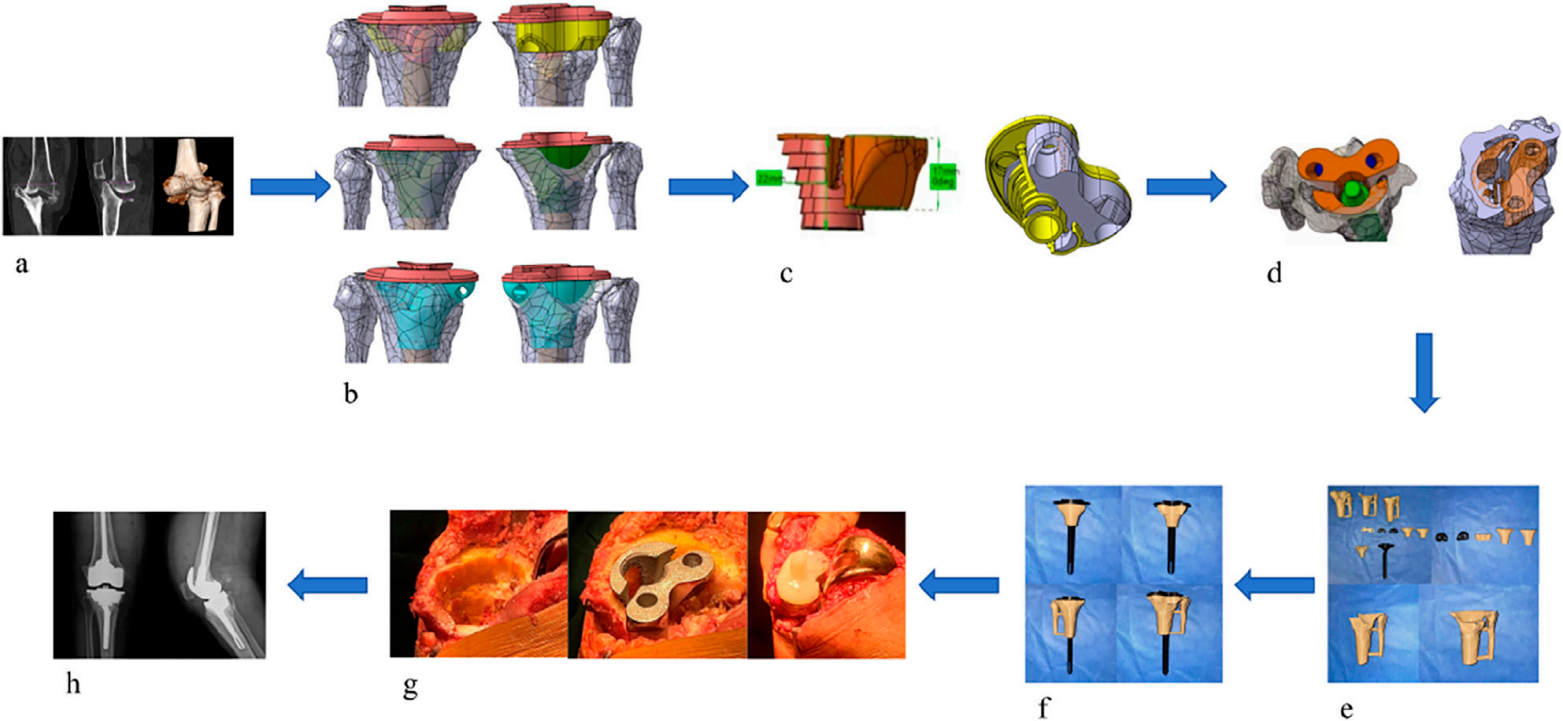
**(a)** Preoperative imaging examinations of the patient. **(b)** Propose different methods based on the patient’s imaging data. **(c)** Select the most suitable method among them. **(d)** Simulate the surgical plan on a computer. **(e)** 3D print a physical model for simulation. **(f)** Validate the Feasibility of the Optimal Method **(g)** Perform the surgical procedure. **(h)** Postoperative imaging examinations.

After obtaining approval from the Institutional Ethics Committee of Zhongshan Hospital Affiliated to Dalian University, a retrospective study was conducted. Six patients with AORI Type III arthritis who underwent total knee arthroplasty (TKA) with 3D-printed custom integrated tibial prosthesis (ITP) at the Knee Joint Department of our hospital from January 2021 to January 2025 were included. There were 2 male and 4 female patients, with an average age of 65.2 ± 8.5 years. The inclusion criteria were as follows: 1) diagnosed with AORI Type III knee osteoarthritis; 2) the patient agreed to knee replacement surgery and signed the informed consent form. The exclusion criteria were: 1) patients under 30 years old or over 80 years old; 2) the primary diseases of the knee joint were other autoimmune diseases, infectious arthritis, or neoplastic diseases. The geometric shape study and analysis of the patients focused on one real surgical case, and a 68-year-old female patient who received 3D-printed integrated prosthesis implantation was selected. Her right knee had suffered from traumatic arthritis due to trauma, resulting in significant collapse of the tibial plateau, accompanied by pain and movement disorders. Preoperative Design and Preparation of the Prosthesis: Before the final design was determined, the preoperative design and preparation work of the prosthesis was carried out first. The CT data of the patient’s knee joint were used for 3D reconstruction through medical imaging processing software. The state of bone defect was simulated based on the real anatomical position of the tibia, and a personalized prosthesis was designed for the medial bone defect area of the tibial plateau. Prosthesis design parameters were consistent with the TKA implantation parameters described in Section 2.2 (0° varus, 1° posterior slope, 3° external rotation) (Dong et al., 2020), ensuring alignment between clinical surgical techniques and finite element model implantation angles. In the early stage of this stage, several sets of bone implant plans were iterated, but all had limitations in clinical application. Some plans had osteotomy amounts exceeding the safe threshold, while others could not effectively fill the bone defect area. After multi-dimensional evaluation, the design was optimized to address the above problems. Through 3D modeling, a connection structure with anatomical matching between the spacer and the tibial plateau was created, and the edges were designed with smooth curved surface transitions. According to the simulation results, the anatomical characteristics of the defect site, and the remaining bone mass, the diameter and thickness of the spacer were precisely optimized to achieve biomechanical adaptation with the host bone. Finally, a design plan with the advantages of maximizing bone defect filling and minimizing osteotomy amount was selected. The integrated prosthesis model data were imported into a 3D printer, and the prosthesis was fabricated by additive manufacturing using the laser powder bed fusion technology with Cobalt-chrome alloy powder as the raw material, realizing the integrated molding construction of the implant and the prosthesis (Figures 2a–f).

#### 2.6.2 Surgical procedure

After successful anesthesia, the patient was placed in the supine position. Routine disinfection and draping were performed, and a median incision was made. The operation was performed according to standard TKA procedures, with key steps including: ① Cleaning the tibial plateau defect area and free bone fragments; ② Implanting the ITP according to preoperative design and adjusting the force line; ③ Fixing the prosthesis after soft tissue balancing. (Figure 2g).

#### 2.6.3 Postoperative management

Postoperative anteroposterior X-ray films of the knee joint showed that the position of the knee joint prosthesis was good and the lower limb alignment was normal (Figure 2h). Antibiotics were infused within 24 h to prevent infection. After anesthesia recovery, the patient was guided to perform ankle and knee flexion and extension exercises and isometric quadriceps muscle strength training. Partial weight-bearing exercise was allowed 24–48 h after surgery, and full weight-bearing exercise was allowed 4 weeks after surgery.

### 2.7 Evaluation criteria

In the systematic assessment of AORI type Ⅲ tibial plateau defect reconstruction, the initial stability of the ITP and the other three bone implants was compared and analyzed by quantifying the contact stress at the interface between the tibial prosthesis and the host bone, the settlement of the tibial plateau, and the relative displacement. Meanwhile, the KSS/KOOS knee joint scoring system was used to clinically evaluate the postoperative knee joint function. Additionally, imaging methods were employed to closely observe key indicators such as radiolucent lines, displacement, aseptic loosening, osteolysis, and bone growth between the prosthesis and the bone surface, establishing a dual evaluation system integrating biomechanics and clinical function. Gait analysis was conducted using the Walkway MW - 1,000 type 2.4 m × 0.6 m sheet - shaped foot pressure - ground gait analyzer from ANIMA Company on a 6.4 m × 0.6 m dedicated walkway (including 2 - meter front and rear auxiliary paths). The subjects completed two walking tests at a comfortable and natural speed. The system collected basic gait parameters, such as walking speed, step length, step width, and cadence, as well as unilateral gait parameters, including stride time, stance time, swing - phase time, double - limb support - phase time, stride, and step length. The research design included a longitudinal comparison of the basic gait information of the preoperative group and the 1 - year postoperative group, as well as a transverse and longitudinal cross - comparison of the basic and unilateral gait parameters of the affected and contralateral sides of the OA group before and 1 year after surgery. This comprehensive assessment of the impact of surgical intervention on the patient’s gait pattern provided an objective and quantitative basis for clinical efficacy evaluation.

## 3 Result

### 3.1 Comparison of contact stress distribution between integral tibial prosthesis (ITP) and other three types of metal augments

**FIGURE 3:**
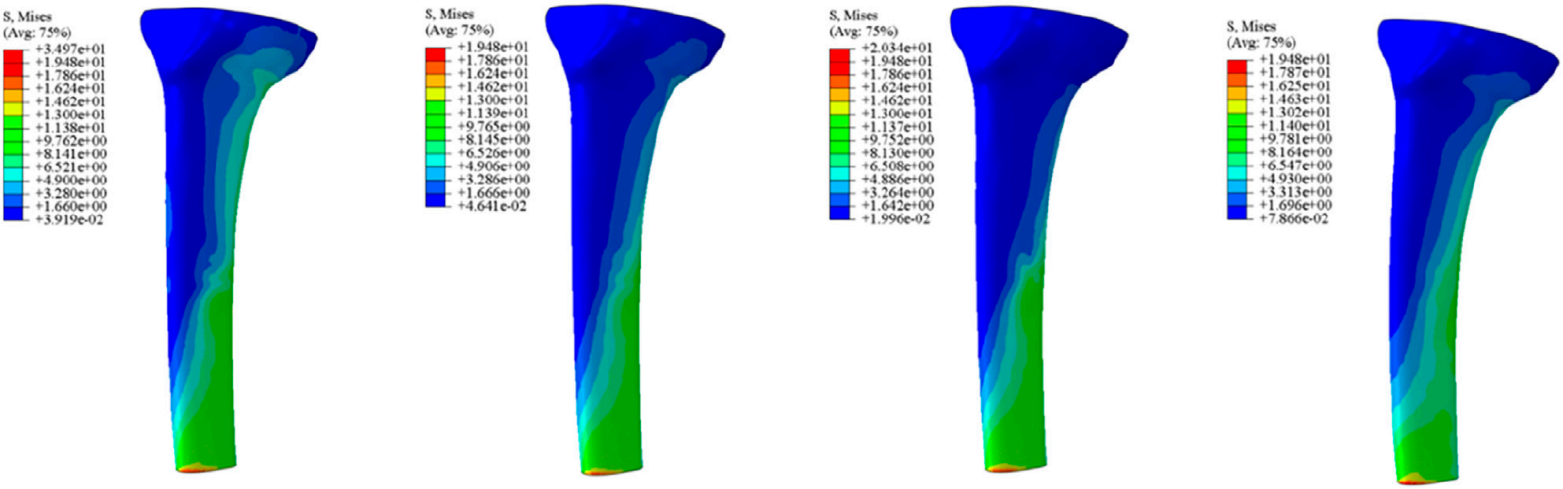
Cortical bone peak stress (MPa). From left to right: spacer, cone, sleeve, ITP.

**FIGURE 4:**
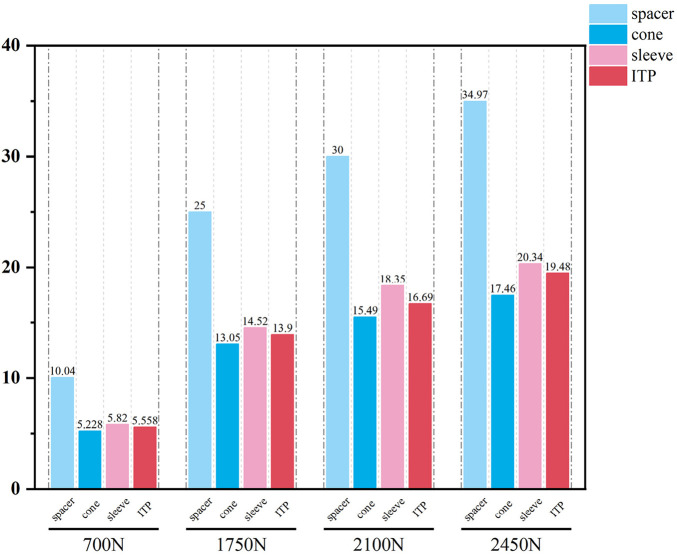
Grouped bar chart of the corticalbone peak stress (MPa).

**FIGURE 5:**
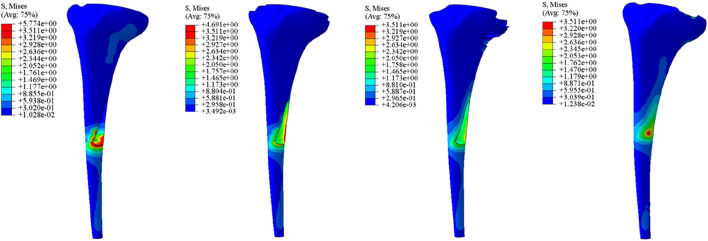
Cancellous bone peak stress (MPa). From left to right: spacer, cone, sleeve, ITP.

**FIGURE 6:**
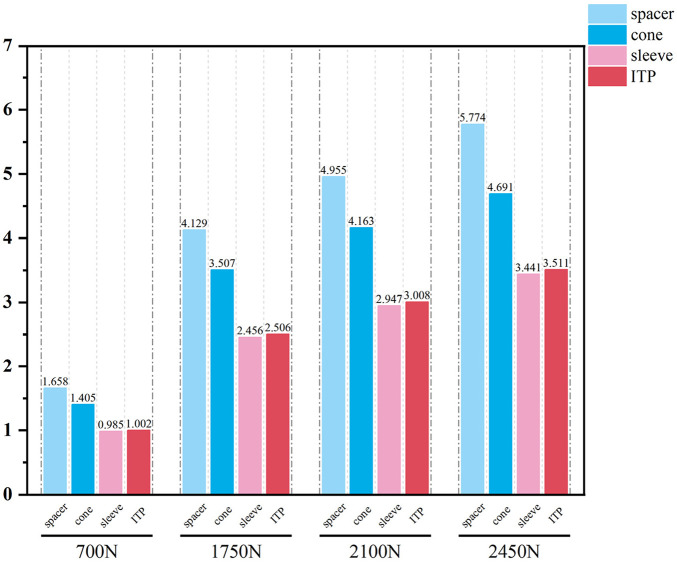
Grouped bar chart of the cancellousbone peak stress (MPa).

**FIGURE 7:**
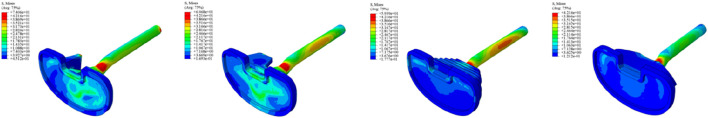
The implants peak stress (MPa). From left to right: spacer, cone, sleeve, ITP.

**FIGURE 8:**
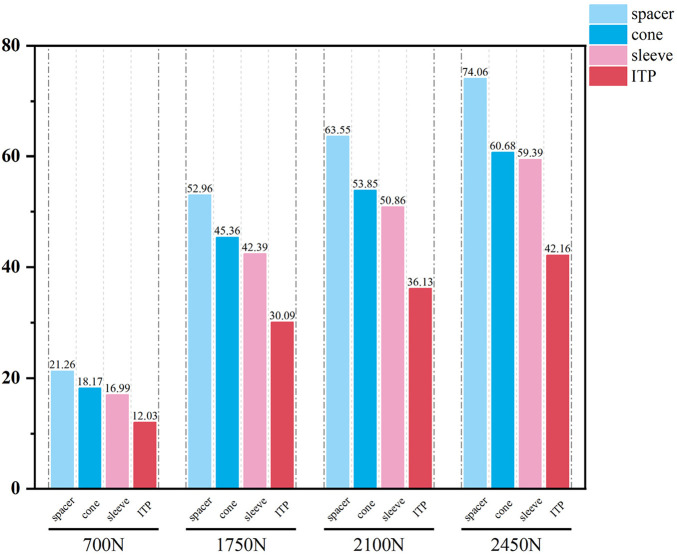
Grouped bar chart of the implants peak stress (MPa).

Initial stability was evaluated by quantifying the contact stress at the prosthesis-bone interface, tibial plateau settlement, and relative micromotion. The contact interface between the prosthesis and bone is divided into cortical bone and cancellous bone. For the peak stress at the cortical bone interface: the maximum stress peaks of ITP at the contacting cortical bone interface are 5.425 MPa (700 N), 13.64 MPa (1750 N), 16.32 MPa (2100 N), and 19.06 MPa (2450 N); those of the spacer are 6.66 MPa (700 N), 16.74 MPa (1750 N), 20.03 MPa (2100 N), and 23.4 MPa (2450 N); those of the cone are 5.716 MPa (700 N), 14.37 MPa (1750 N), 17.19 MPa (2100 N), and 20.08 MPa (2450 N); those of the sleeve are 5.734 MPa (700 N), 14.44 MPa (1750 N), 17.27 MPa (2100 N), and 20.18 MPa (2450 N); the maximum stress concentration point is at the distal end of the cortical bone. For the maximum stress peaks at the contacting cancellous bone interface: those of ITP are 0.868 MPa (700 N), 2.182 MPa (1750 N), 2.612 MPa (2100 N), and 3.050 MPa (2450 N); those of the spacer are 1.211 MPa (700 N), 3.044 MPa (1750 N), 3.645 MPa (2100 N), and 4.255 MPa (2450 N); those of the cone are 1.080 MPa (700 N), 2.714 MPa (1750 N), 3.249 MPa (2100 N), and 3.794 MPa (2450 N); those of the sleeve are 1.118 MPa (700 N), 2.810 MPa (1750 N), 3.364 MPa (2100 N), and 3.928 MPa (2450 N); the maximum stress concentration point is at the contact area between the cancellous bone and the distal end of the extension stem. For the peak stresses at the prosthesis interface: those of ITP are 25.59 MPa (700 N), 64.31 MPa (1750 N), 77.01 MPa (2100 N), and 89.9 MPa (2450 N), with the stress concentration point at the corner of the extension stem; those of the spacer are 18.82 MPa (700 N), 47.3 MPa (1750 N), 56.63 MPa (2100 N), and 66.12 MPa (2450 N); those of the cone are 19.17 MPa (700 N), 48.19 MPa (1750 N), 57.69 MPa (2100 N), and 67.37 MPa (2450 N); those of the sleeve are 30.62 MPa (700 N), 76.67 MPa (1750 N), 92.18 MPa (2100 N), and 107.60 MPa (2450 N); the maximum stress concentration is at the corner of the extension stem (Figures 3–8).

### 3.2 Comparison of the relative micromotion relative to the host bone between ITP and the other three metal augments

**FIGURE 9:**
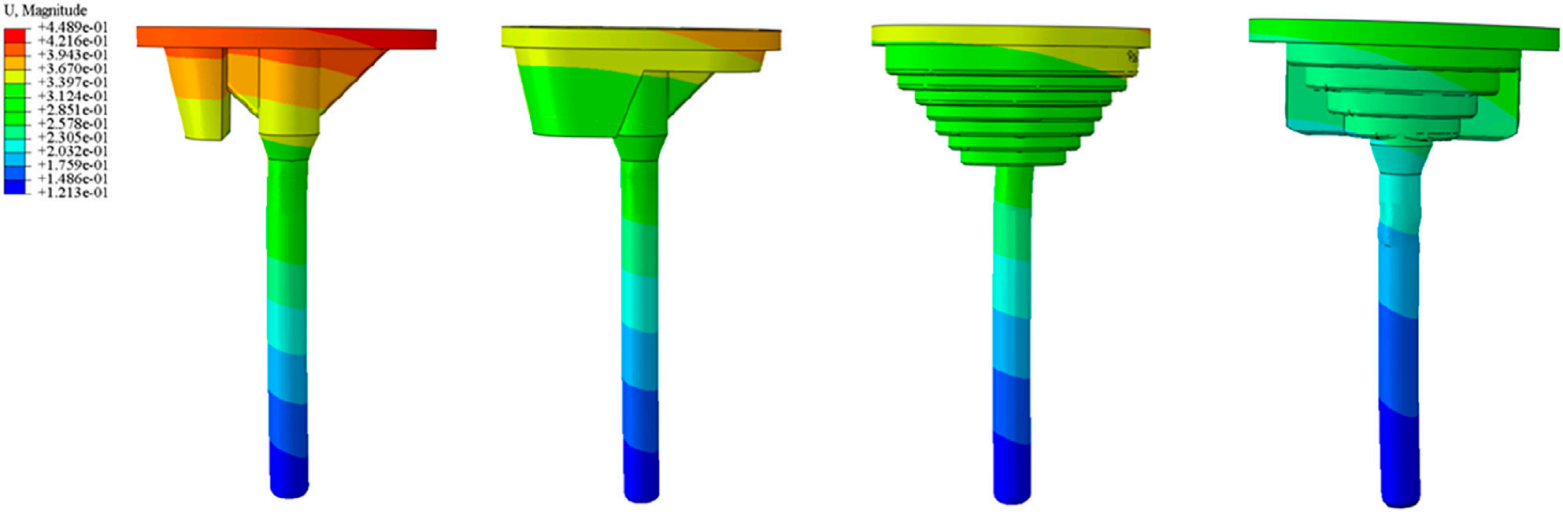
Tibial Plateau Settlement (μm). From left to right: spacer, cone, sleeve, ITP.

**FIGURE 10:**
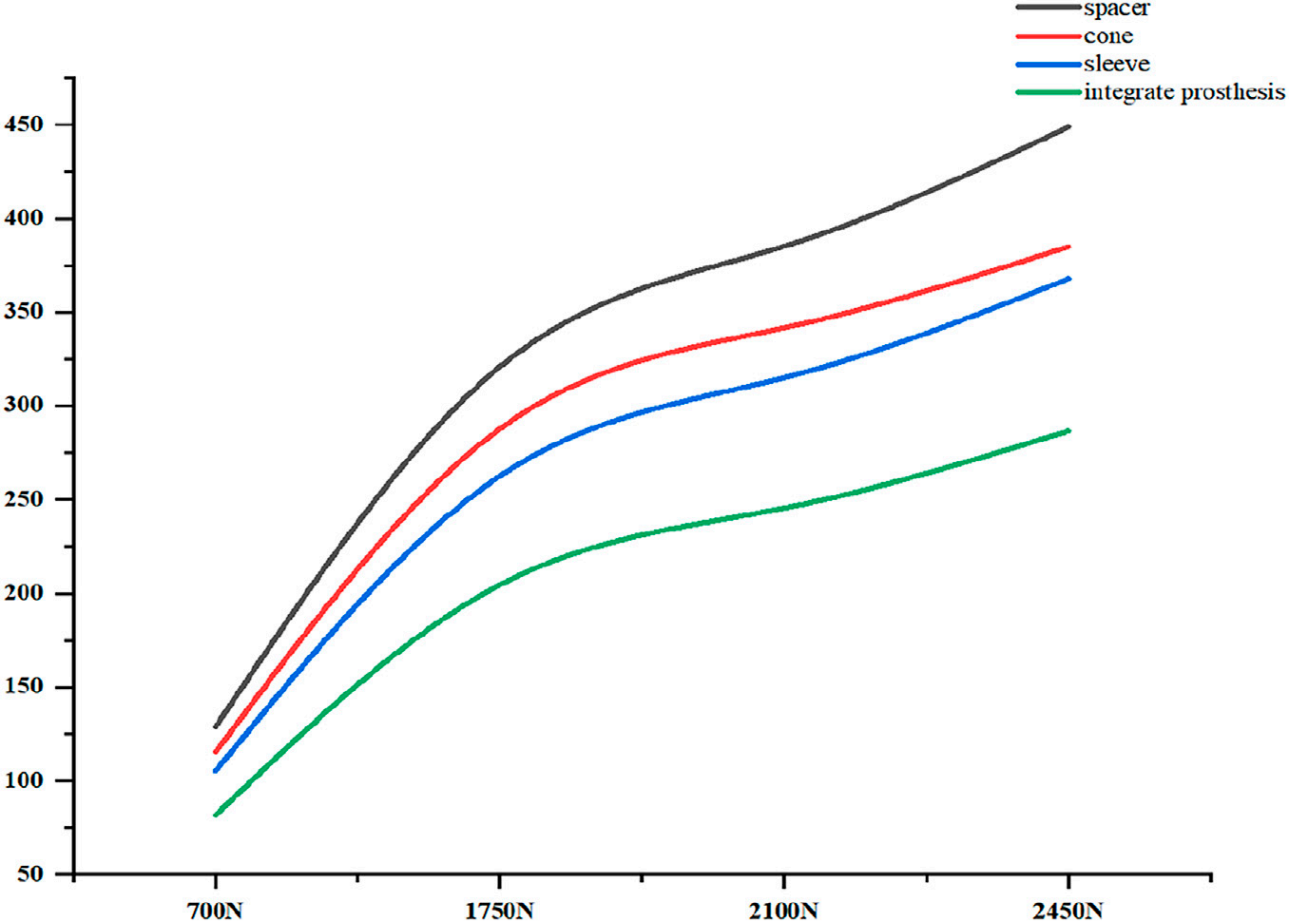
Curve Chart of Tibial plateau settlement (μm).

**FIGURE 11:**
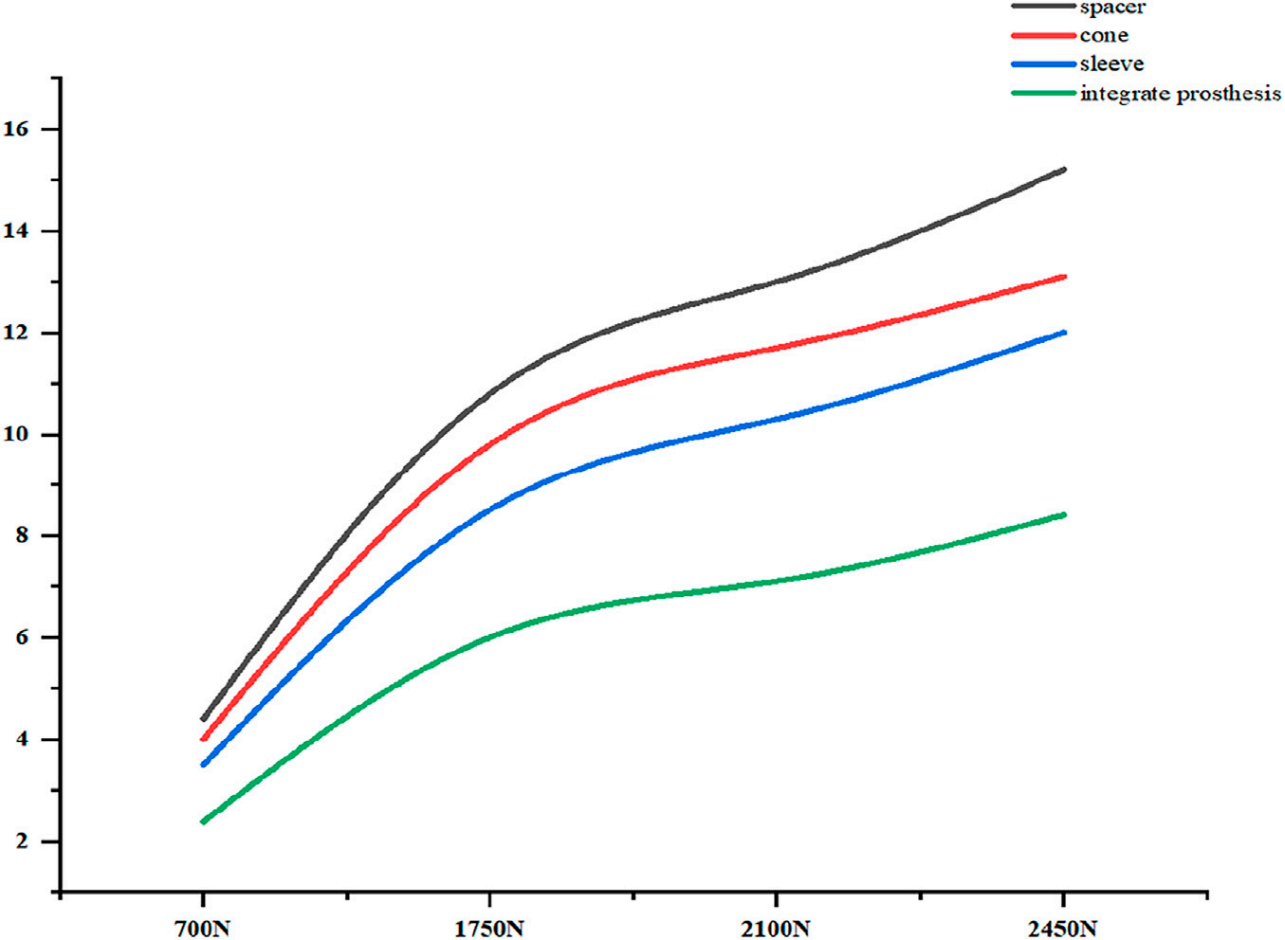
Relative micromotion peak (μm).

The relative micromotion between the tibial prosthesis and the tibia—defined as the displacement difference between two adjacent nodes (one on the bone and one on the tibial plateau)—were recorded. For the ITP, the peak micromotions relative to the host bone were 15.2 μm (700 N), 38.3 μm (1750 N), 45.7 μm (2100 N), and 53.6 μm (2450 N). For the spacer, the peak micromotions relative to the host bone were 16.6 μm (700 N), 41.7 μm (1750 N), 49.9 μm (2100 N), and 58.3 μm (2450 N). For the cone, the peak micromotions relative to the host bone were 19.17 μm (700 N), 48.19 μm (1750 N), 57.69 μm (2100 N), and 67.37 μm (2450 N). For the sleeve, the peak micromotions relative to the host bone were 17.8 μm (700 N), 44.6 μm (1750 N), 53.3 μm (2100 N), and 62.3 μm (2450 N), respectively (Figures 9–11).

### 3.3 Patient clinical follow-up results

**FIGURE 12:**
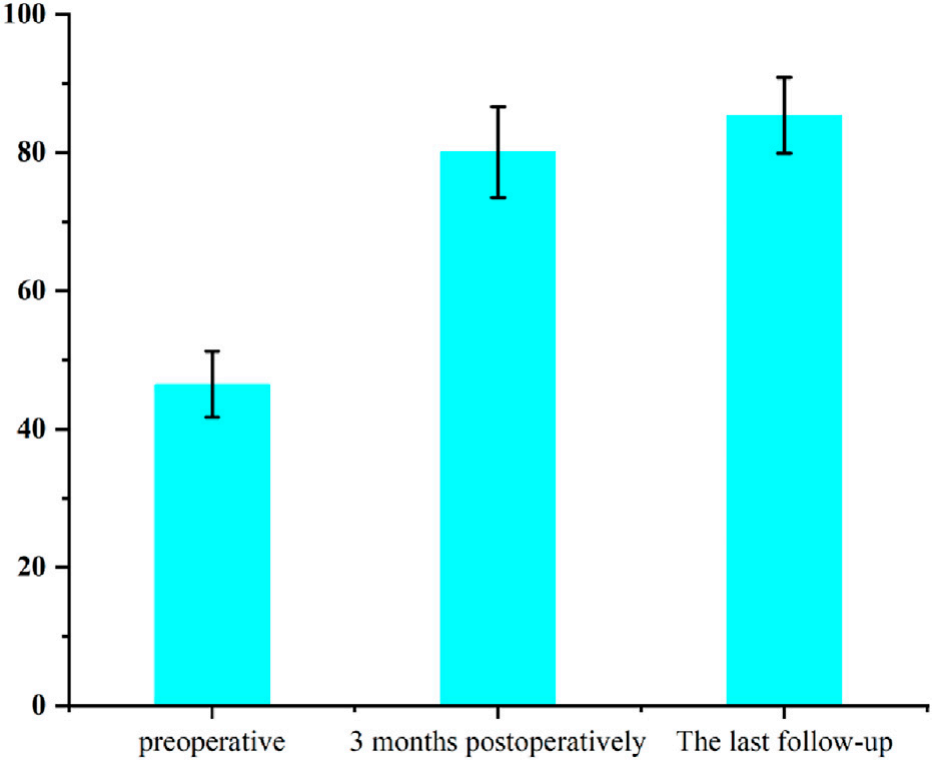
Patient’s KSS score.

**FIGURE 13:**
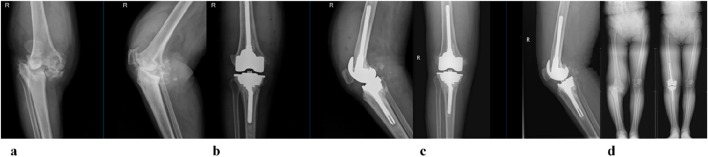
A 65-year-old female patient with AORI Type III post-traumatic knee arthritis (PTKA) underwent 3D-printed integrated trabecular prosthesis (ITP) knee reconstruction. **(a)** Preoperative X-ray; **(b)** X-ray at 1 week post-surgery; **(c)** X-ray at the final follow-up; **(d)** Comparison of preoperative and postoperative lower extremity alignment.

Clinical functional recovery was evaluated using the KSS scoring system, X-ray imaging to assess prosthesis position and bone integration, and gait analysis.The patient was followed up for 24 months. The preoperative KSS knee score was 46.5 ± 4.8. It was 80.1 ± 6.6 at 3 months postoperatively and 85.4 ± 5.5 at the last follow-up (Figure 12). At the last follow-up, there was no swelling, infection or postoperative complications at the surgical site. X-rays of the knee joint in anteroposterior position of all patients showed no adverse conditions such as radiolucency, loosening, or osteolysis around the 3D-printed ITP and bone surface (Figures 13A–C).

### 3.4 The results of the patient’s postoperative gait analysis

In terms of basic gait information, the preoperative mean walking speed was 0.49 m/s, with step lengths of 45.4 cm, cadences of 85.8 steps/min, and step widths of 10.3 cm. At 1 year postoperatively, the mean walking speed increased to 1.08 m/s, step lengths increased to 52.6 cm, cadences rose to 122.8 steps/min, while there was no significant improvement in step widths. In the unilateral gait information, the preoperative stance times of the contralateral and affected sides were 0.71 s and 0.68 s, respectively; the swing phase times were 0.35 s and 0.40 s, respectively; the double-limb support phase times were 0.18 s and 0.17 s, respectively. Both sides had a stride time of 1.07 s, a stride of 88.0 cm, and step lengths of 45.4 cm and 45.9 cm, respectively. At 1 year postoperatively, the stance time, swing phase time, and double-limb support phase time of the contralateral and affected sides tended to be consistent, being 0.61 s, 0.36 s, and 0.12 s, respectively. Both sides had a stride time of 1.00 s, a stride increased to 105.6 cm, and step lengths of 52.7 cm and 52.6 cm, respectively (Table 2).

**TABLE 2:** Gait parameters comparison preoperatively, 1-year postoperatively, and between contralateral and affected sides.

Basic gait information	Preoperative	1 year postoperatively
mean walking speeds (m/s)	0.49 ± 0.06	1.08 ± 0.09
step lengths (cm)	45.4 ± 2.1	52.6 ± 2.3
cadences (steps/min)	85.8 ± 4.2	122.8 ± 5.1
step widths (cm)	10.3 ± 0.8	No significant improvement

Unilateral gait information	Contralateral side	Affected side	Contralateral side	Affected side
stance time(s)	0.71 ± 0.03	0.68 ± 0.04	0.61 ± 0.02	0.61 ± 0.02
the swing phase times(s)	0.35 ± 0.02	0.40 ± 0.03	0.36 ± 0.04	0.36 ± 0.04
double-limb support phase time(s)	0.18 ± 0.01	0.17 ± 0.01	0.13 ± 0.01	0.13 ± 0.01
stride time(s)	1.07 ± 0.03	1.07 ± 0.03	1.01 ± 0.02	1.01 ± 0.02
stance time(s)	0.73 ± 0.12	0.69 ± 0.11	0.62 ± 0.10	0.62 ± 0.10
stride (cm)	88.0 ± 3.91	88.0 ± 3.91	105.6 ± 2.15	105.6 ± 2.15
step lengths (cm)	45.4 ± 3.3	45.9 ± 3.3	52.7 ± 2.7	52.6 ± 2.7

It should be noted that the results of this study are applicable to large-area non-contained AORI, Type Ⅲ defects (defect area >60% and depth >20 mm). For smaller subtypes (e.g., defect area loss of 40%–60%), the stress advantages of ITP, may be weakened, but they remain meaningful.

## 4 Discussion

The high 10-year revision rate (23%) of traditional modular augment prostheses (MAP) in treating AORI Type Ⅲ tibial plateau defects underscore the urgent need for improved reconstruction strategies (Passias et al., 2020). These clinical dilemmas—rooted in extensive metaphyseal bone loss, cortical discontinuity, and biomechanical mismatches of standardized components—provided the impetus for our study comparing 3D-printed integrated tibial prostheses (ITP) and conventional MAP. Our findings, from both finite element analysis and preliminary clinical follow-up, offer new insights into addressing these long-standing challenges.

Finite element results revealed that ITP outperformed three MAP subtypes (Spacer, Cone, Sleeve) across all load conditions (700N–2450N), particularly in interface stress distribution and micromotion control. Under 2100N loading (simulating stair climbing), ITP reduced peak cortical bone stress by 45% (16.69 MPa vs 30.00 MPa in Spacer group) and relative micromotion by 45% (7.1 μm vs 13.0 μm), directly addressing the two key drivers of aseptic loosening: excessive stress-induced bone resorption and micromotion exceeding critical thresholds (Jyoti et al., 2022). These biomechanical advantages, we argue, stem from ITP’s design innovations rather than material differences—both ITP and MAP in our study utilized cobalt-chrome alloy, eliminating material as a confounding variable.

The biomechanical superiority of ITP derives from two core design features. First, its integrated structure eliminates modular interfaces, a inherent weakness in MAP. Traditional MAP relies on assembled components (tibial tray + augment + stem), where interface micromotion generates wear particles (e.g., cobalt-chrome debris) that trigger inflammatory responses and periprosthetic osteolysis—consistent with Clatworthy et al. (2001) observations of stress concentration and osteolysis at stem-distal interfaces in MAP users (Parks et al., 1998). ITP’s one-piece design avoids this risk entirely. Second, ITP’s patient-specific morphological matching (via preoperative CT/MRI 3D reconstruction) ensures precise adaptation to irregular AORI Type Ⅲ defects, unlike standardized MAP, which requires intraoperative adjustments (e.g., repeated cone repositioning). Such adjustments prolong surgery by ∼25 min and increase infection risk by 1.8-fold (Lei et al., 2020; Coelho et al., 2024), whereas ITP’s prefabricated design achieves immediate, uniform defect coverage.

Clinical evidence from 24-month follow-up of 6 patients further supports ITP’s value. The Knee Society Score (KSS) improved from 46.5 ± 4.8 preoperatively to 85.4 ± 5.5, exceeding the “good recovery” threshold (KSS >80). Radiologically, no periprosthetic radiolucency, loosening, or osteolysis was observed (Figure 12), aligning with Zerbo et al. (2003) findings that conforming prosthetic structures promote bone integration through enhanced osteocyte migration and vascular ingrowth (Zerbo et al., 2003). Gait analysis confirmed functional recovery: 1-year postoperative walking speed reached 1.08 m/s (meeting Studenski et al. (2011) “good functional status” standard) (Studenski et al., 2011), step length increased by 15.8% (45.4 cm–52.6 cm), and stance/swing-phase symmetry approached that of the contralateral limb—outcomes rarely achieved with MAP due to imprecise defect filling.

To contextualize these findings, it is critical to highlight the limitations of traditional repair techniques that ITP addresses. Autologous bone grafting, while biologically integrable, suffers 40% bone resorption at 5 years and donor-site complications (Wallace et al., 2020). Cement-screw fixation induces stress shielding due to elastic modulus mismatches (2270 MPa for cement vs 700–17000 MPa for bone), leading to subsidence (Kendrick et al., 2015). Modular metal augments, despite widespread use, fail to match complex defect morphologies, requiring intraoperative adjustments that elevate infection risk (Denehy et al., 2019; Rodríguez-Merchán, 2021). Our finite element data further demonstrate that under 2450N loading, metal augments exhibit 64.5% higher peak interface stress (5.774 MPa) than ITP, confirming their biomechanical inferiority.

Notwithstanding these promising results, our study has limitations. The finite element model excluded ligaments, menisci, and muscle forces, which influence knee biomechanics during dynamic movements (e.g., squatting). Future models should integrate a complete ligament-muscle system to simulate realistic loading. Second, we tested only one AORI Type Ⅲ subtype (area >60%, depth >20 mm); expanding to smaller or combined medial-lateral defects will enhance generalizability. Third, the single-arm design (n = 6) and lack of MAP controls limit causal inference. Multicenter randomized controlled trials (RCTs) comparing 5-year revision rates, WOMAC scores, and radiological bone integration are needed to validate long-term efficacy. Specifically, future multicenter randomized controlled trials (RCTs) should be conducted to compare the long-term efficacy of ITP and MAP (e.g., 5-year prosthesis survival rates, revision rates) in order to validate the clinical advantages of ITP. Another limitation is the mismatch in bone quality between the finite element model and clinical patients: the model was built based on a 24-year-old healthy male, while the enrolled patients had an average age of 65.2 ± 8.5 years and may have had osteoporosis, which could potentially lead to deviations in the model’s calculated biomechanical results (e.g., underestimating actual prosthesis-bone interface stress or overestimating bone deformation resistance).

## 5 Conclusion

AORI type III tibial plateau defects in TKA and rTKA remain challenging, with traditional techniques limited by high loosening, revision rates, and biomechanical mismatches. 3D-printed integrated prostheses address these via personalized matching and biomechanical optimization, improving function and bone integration. While promising short-term, they are limited by single-arm design (n = 6, no MAP controls) and need future multicenter RCTs for long-term validation become the standard for such defects.

## Data Availability

The original contributions presented in the study are included in the article/supplementary material, further inquiries can be directed to the corresponding author.
